# Female‐induced selective modification of sperm protein SUMOylation—potential mechanistic insights into the non‐random fertilization in humans

**DOI:** 10.1111/jeb.13980

**Published:** 2022-01-19

**Authors:** Jukka Kekäläinen, Johannes Hiltunen, Annalaura Jokiniemi, Liisa Kuusipalo, Marjo Heikura, Jonna Leppänen, Marjo Malinen

**Affiliations:** ^1^ Department of Environmental and Biological Sciences University of Eastern Finland Joensuu Finland; ^2^ North Karelia Central Hospital Joensuu Finland; ^3^ Eastern Finland Laboratory Centre Joint Authority (ISLAB) North Savonia Regional Laboratory Kuopio Finland; ^4^ Kuopio University Hospital Kuopio Finland

**Keywords:** cryptic female choice, fertilization, follicular fluid, mate choice, protein, sperm, SUMOylation

## Abstract

In many species, mate choice continues after the mating via female‐ or egg‐derived biochemical factors that induce selective changes in sperm pre‐fertilization physiology and behaviour. Recent studies have indicated that gamete‐mediated mate choice likely occurs also in humans, but the mechanistic basis of the process has remained virtually unexplored. Here, we investigated whether female‐induced modifications in sperm protein SUMOylation (post‐translational modification of the proteome) could serve as a novel mechanism for gamete‐mediated mate choice in humans. We treated the sperm of ten males with the oocyte‐surrounding bioactive liquid (follicular fluid) of five females and investigated motility, viability and global protein SUMOylation status of the sperm in all (*n* = 50) of these male–female combinations (full‐factorial design). All the measured sperm traits were affected by male–female combinations, and sperm protein SUMOylation status was also negatively associated with sperm motility. Furthermore, our results indicate that female‐induced sperm protein SUMOylation is selective, potentially allowing females to increase sperm motility in some males, whereas decreasing it in the others. Consequently, our findings suggest that follicular fluid may non‐randomly modify the structure and function of sperm proteome and in this way facilitate gamete‐mediated mate choice in humans and possibly many other species. However, due to the relatively low number of female subjects and their potential infertility problems, our results should be replicated with larger subset of fully fertile women.

## Graphical abstract

In many species, mate choice continues after the mating at the level of the gametes. However, the mechanistic basis of gamete‐mediated mate choice has remained largely unclear. Here we investigated whether female‐induced post‐translational modifications in sperm proteome could serve as a novel mechanism for gamete‐mediated mate choice in humans.

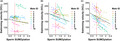

## 1 INTRODUCTION

Fertilization is critically dependent on the chemical signals released from the unfertilized oocytes and (in internally fertilizing species) the reproductive tract of the female (Eisenbach & Giojalas, 2006; Rickard & de Graaf, 2020). These female‐derived signals induce a number of physiological responses in sperm, such as capacitation (‘sperm maturation’), hyperactivation, acrosome reaction and guide sperm towards oocytes (chemotaxis) (Duan et al., 2020; Kekäläinen et al., 2015; Pitnick et al., 2020; Yoshida et al., 2008). Earlier studies in externally fertilizing species have shown that female‐derived chemical signals may have an additional function in gamete‐mediated mate choice and thus potentially facilitate cryptic female choice towards genetically compatible (or otherwise preferred) males (Alonzo et al., 2016; Evans et al., 2012; Geßner et al., 2017; Lymbery et al., 2017 Rosengrave et al., 2016, reviewed by Kekäläinen & Evans, 2018). On the contrary, experimental evidence for gamete‐mediated mate choice in internally fertilizing species has been extremely limited (but see Gasparini & Pilastro, 2011) and it has been unclear whether cryptic female choice could occur in humans. However, recent studies have indicated that female (i.e. women's) reproductive tract (FRT) secretions (cervical mucus and follicular fluid) may have a key role in facilitating partner selection at the level of the gametes (Fitzpatrick et al., 2020; Jokiniemi, Kuusipalo, et al., 2020; Jokiniemi, Magris, et al., 2020). Nevertheless, molecular‐level mechanisms of the findings—and gamete‐mediated mate choice in general—have remained elusive (Kekäläinen & Evans, 2017; Lüpold et al., 2020; Manier et al., 2013).

Freshly ejaculated mammalian spermatozoa are incapable of fertilizing the oocyte, until acquiring fertilization competence in FRT (Nixon et al., 2020; Saint‐Dizier et al., 2020). Given that mature spermatozoa are generally thought to be transcriptionally and translationally silent cells, protein post‐translational modifications (PTMs) are believed to play a vital role in regulating sperm function (Marchiani et al., 2014; Martin‐Hidalgo et al., 2020; Ritagliati et al., 2018; Samanta et al., 2016; Schon et al., 2019). PTMs refer to various enzymatic alterations in protein structure following their biosynthesis, which increase the diversity of proteome and regulate both protein stability and function (Samanta et al., 2016). In sperm, PTMs have been shown to modify sperm maturation and acquisition of fertilizing potential in FRT (Castillo et al., 2019). More than 300 types of PTMs have been identified, but currently, only a minor subset of the PTMs have been thoroughly studied (Baker, 2016). Earlier studies have shown that sperm proteins are commonly modified by phosphorylation and nitrosylation (Holt & Fazeli, 2015), whereas large majority of other PTMs, such as SUMOylation, have remained much less studied.

SUMOylation is a PTM in which Small Ubiquitin‐like Modifier (SUMO) proteins are reversibly attached to cellular proteins (Samanta et al., 2016). In humans, SUMO proteins exist in four isoforms and three of them (SUMO 1–3) have been detected in human sperm. SUMO 2 and SUMO 3 are 95% identical and are thus often referred to as SUMO 2/3 (Vigodner et al., 2013). SUMOylation is known to have an important role in many cellular‐level processes, such as DNA damage control and regulation of mitochondrial dynamics (Marchiani et al., 2014). However, despite the fact that SUMOylation has been extensively studied in somatic cells, only few earlier studies have identified this PTM in mature sperm (Marchiani et al., 2014). Excessive SUMOylation of human sperm has been demonstrated to indicate poor sperm quality, such as defective morphology and low motility (Marchiani et al., 2014; Samanta et al., 2016; Vigodner et al., 2013). Marchiani et al. (2014) also showed that SUMOylation may increase in mature sperm cells in stressful conditions and reduce the integrity and functionality of sperm mitochondria. Given that functional mitochondria are vital for sperm motility, this finding provides a potential mechanistic explanation why excessive SUMOylation of sperm proteins has been found to be associated with impaired sperm function. Together, these findings indicate that SUMOylation of sperm proteins act as an important regulator of sperm fertilization success and male fertility (Rodriguez & Pangas, 2016).

After copulation, sperm motility and fertilization capability are critically dependent on the secretions of the FRT, such as follicular fluid (Saint‐Dizier et al., 2020; Suarez & Pacey, 2006). Jokiniemi, Kuusipalo, et al. (2020) recently demonstrated that various sperm physiological responses (motility, hyperactivation, acrosome reaction and viability) to follicular fluid are strongly dependent on male–female interaction (combination) and human leucocyte antigen (HLA) genotype of the partners. It has also been shown that follicular fluid contains sperm chemoattractants that selectively attracts sperm of specific males over others (Fitzpatrick et al., 2020). Together, these two studies indicate that follicular fluid facilitates gamete‐mediated mate choice towards compatible partners in humans. However, the mechanistic basis of this cellular‐level mate choice has remained elusive (reviewed by Kekäläinen & Evans, 2018). Kekäläinen and Evans (2017) recently demonstrated in the externally fertilizing marine mussel *Mytilus galloprovincialis* that egg‐derived chemical factors trigger structural changes in sperm surface glycosylation (one type of PTM). Furthermore, sperm glycosylation was strongly dependent on male–female combination and possibly regulated sperm intracellular Ca^2+^ concentration and associated pre‐fertilization physiological changes in sperm. Together, these results indicate that sperm PTMs may have some currently unknown function in cryptic female choice, although the role of PTMs in post‐mating sexual selection has remained virtually unexplored (but see Ghaderi et al., 2011).

In the present study, we investigated whether follicular fluid‐induced changes in the sperm protein SUMOylation have the potential to facilitate cryptic female choice in humans. We treated the sperm of 10 men with the follicular fluid of five women, in all possible male–female combinations (full‐factorial design: *n* = 50 combinations) and measured motility, hyperactivation and viability of sperm in all these combinations. Then, we investigated the effect of follicular fluid on global sperm protein SUMOylation (SUMO 2/3) in each independent male–female combination by Western blot. Finally, we tested whether sperm SUMOylation status predicts sperm motility and viability in the follicular fluid. Based on the demonstrated importance of protein SUMOylation for sperm function, we predicted that follicular fluid‐induced changes in SUMOylation of sperm proteins provides novel mechanistic insights into the female‐mediated sperm selection in humans.

## 2 MATERIAL AND METHODS

### 2.1 Study subjects and sample collection

Female participants (*n* = 5) in this study were recruited via the fertility clinics of Kuopio University Hospital and North Karelia Central Hospital (Finland) in January–April 2018. Three of the women did not have any infertility diagnosis. The remaining two women had been diagnosed with ovulation disorder and polycystic ovary syndrome respectively. Four of the women have biological child and fifth woman have had clinical pregnancy. The mean age for the participating women was 32.6 (range 26–38, ±1.94 SE) years. Follicular fluid samples were collected from females undergoing transvaginal follicular aspiration for *in vitro* fertilization. Before collection, follicle maturation was hyperstimulated with follicle‐stimulating hormone (FSH) and premature ovulation was prevented using a gonadotrophin‐releasing hormone antagonist (GnRH). When the diameter of the largest follicle reached 18–20 mm, human chorion gonadotrophin (hCG) was administered, and follicles were collected. A transvaginal follicular puncture was performed under local anaesthesia, using ultrasound guidance. After collection, follicular fluid samples were centrifuged at 500×*g* for 10 min, and the supernatant was aliquoted and stored in liquid nitrogen for later use.

Male participants (*n* = 10) were recruited from the fertility clinic of North Karelia Central Hospital and through advertisements in the University of Eastern Finland's internal information channels. All the males had normal sperm motility and sperm count according to World Health Organization (WHO) criteria. The mean age for the participating men was 30.1 (range 24–38, ±1.73 SE) years. All the men provided semen samples by masturbation after 2–3 days of sexual abstinence. After collection, semen samples were first allowed to liquefy for 30 min at +37°C. To separate mature spermatozoa, the liquefied samples were washed with two‐layer (40% and 80%) density gradient centrifugation (PureSperm^®^ 40 and 80, Nidacon International AB, Mölndal, Sweden), according to manufacturer's protocol. After the density gradient centrifugation, spermatozoa were rinsed by additional centrifugation in PureSperm^®^ Wash solution (Nidacon). We standardized sperm density among all 10 males to the final concentration of ca. 42 (± 2.3 SE) million cells/ml. Before participation in this study, an informed written consent was asked from all the subjects (females and males).

### 2.2 Follicular fluid treatments of the sperm

Follicular fluid of each of the five women was divided in two sub‐samples (A and B: 10 samples in total) and then combined (1:1, volume: volume) with the washed sperm aliquots (see above) from all the 10 males, resulting in 100 follicular fluid‐treated samples (5 females ×10 males × 2 sub‐samples). This 5 × 10 design was selected due to the limited sperm protein concentration that prevented us to subdivide sperm of each male to more than five females in our western blot analyses (see below). Furthermore, two sperm aliquots in each of the 10 males acted as untreated (diluted in PureSperm^®^ Wash solution) control samples (*n* = 120 sperm samples in total). Due to the between‐male variation in total sperm count, and the fact that we standardized the sperm density to similar final concentration (see above), total incubation volumes of sperm samples varied between 110 µl and 460 µl. However, the sperm‐follicular fluid ratio always remained the same (1:1, v:v). All the samples were kept at +37°C (by a warming stage) during the entire experimental period and during the sperm analyses. Furthermore, all the sperm treatments and measurements (see below) were always performed on the day of semen collection (i.e. using fresh sperm).

To minimize a potential time effect on the measured sperm traits, both the initiation of follicular fluid treatments and subsequent sperm measurements (see below) in the first sub‐sample (A) were always conducted in the following order (with 3 min intervals): control, FF1, FF2,…, FF5, whereas in the second sub‐sample (B), initiation of treatment and sperm measurements was performed in the opposite order: FF5, FF4,…, control (see Jokiniemi, Magris, et al., 2020).

### 2.3 Sperm motility and viability measurements

Sperm motility was recorded by adding 1µl of each follicular fluid‐treated sperm sample to pre‐warmed (+37°C) Leja 4‐chamber (chamber height 20 μm) microscope slides (Leja, Nieuw‐Vennep, the Netherlands). Then, sperm motility (curvilinear velocity: VCL; linearity of the swimming trajectory: LIN; and amplitude of the lateral head displacement: ALH) was recorded for one second using computer‐assisted sperm analysis (CASA; Integrated Semen Analysis System, ISAS v. 1.2, Proiser, Valencia, Spain), with a negative phase‐contrast microscope (100 × magnification) and a capture rate of 100 frames/s. Furthermore, following the criteria from Kay and Robertson (1998), the hyperactivated state of the sperm was determined based on three CASA parameters: VCL >150 µm/s, LIN <50% and ALH >2.0. Sperm motility was measured at four time points: 30, 90, 180 and 300 min since the beginning of the follicular fluid treatment. Selection of time points is based on earlier observations on the average duration of sperm motility period and capacitated state of human sperm (ca. 50–240 min) in vitro (Eisenbach & Tur‐Kaspa, 1999). Motility measurements included two replicate chambers from both sub‐samples (performed in two different Leja‐slides) in each of the 50 male–female combinations (resulting in four measurement chambers in total). Furthermore, within each of the four chambers, sperm motility was recorded from at least two independent fields of view. Sperm motility was measured for an average of 2 762 ± 52 (mean ± SE) sperm cells, per male–female combination. All the sperm motility analyses were performed following the most recent standards by the World Health Organization (World Health Organization, 2021).

At the end of the motility measurements, a 5 µl aliquot from all 100 follicular fluid‐treated sperm samples were separated for sperm viability assay. To achieve an optimal sperm concentration for viability measurements, the sample volume was adjusted to the final volume of 25 µl by adding 20 µl of PureSperm^®^ Wash solution‐follicular fluid mixture. After the dilution, sperm were stained with propidium iodide (PI, 5 µg/ml) and incubated for three minutes (in the dark). Then, 0.5 µl of 1% formalin was added to immobilize the sperm and numbers of dead and total sperm cells were immediately measured using a LUNA‐FL™ Dual Fluorescence Cell Counter (Logos Biosystems, Annandale, VA, USA). As described above, sperm viability measurements also included two replicate recordings from both sub‐samples. The proportion of dead cells was measured for an average of 3 363 ± 101 (mean ± SE) sperm cells, per male–female combination.

Finally, after 5 h follicular fluid treatment, the remaining cells from both sub‐samples (A+B) of both follicular fluid‐treated and control samples were combined and pelleted by centrifugation (1500×*g*, for 5 min). The supernatant was discarded, and the pellets were washed with 1 ml of PBS (centrifugation in 1500 × g, for 5 min). Sperm pellets were resuspended in Laemmli buffer (4% SDS, 20% glycerol, 0.125 M Tris‐CL, pH 6.8) with protease inhibitor and N‐Ethylmaleimide and stored in the freezer (−80°C) for later western blot analyses.

### 2.4 Western blot analyses of sperm and follicular fluid SUMO 2/3 expression

Stored sperm pellets were thawed and sonicated (20% amplitude, 0.5 frequency, 2 × 10 s), and protein concentration of the samples was determined by BCA protein assay using bovine serum albumin as a standard (Pierce, Rockford, IL, USA). Before protein electrophoresis, β‐mercaptoethanol (5%) and bromphenol blue (0.02%) were added and the samples were then boiled at 95°C for 5 min. Thawed follicular fluid samples were prepared similarly, except that before sonication and BCA protein assay, the fluids were diluted to 1:10 (v:v) with 1x PBS. Protein electrophoresis for all the sperm samples (control and FF‐treated sperm) within each of the 10 males was conducted in the same gel (two replicate gels/male). Similarly, all five follicular fluid samples were placed in the same gel and protein electrophoresis was replicated in two independent gels. All protein electrophoresis analyses and following Western blotting were performed according to standard procedures. Briefly, based on BCA protein assay (see above), aliquots of sperm and follicular fluid lysates containing equal concentration (10 µg for sperm samples and 5 µg for follicular fluid samples) of proteins were separated on 4–15% gradient mini‐PROTEAN Stain‐Free TGX Precast Gel (Bio‐Rad Laboratories). After electrophoresis, protein concentrations of the wells were detected by ChemiDoc instrument (Bio‐Rad Laboratories) according to manufacturer's instructions for normalization, that is SUMOylation signal of each well was normalized to the total protein concentration of the well. The proteins were transferred to a nitrocellulose membrane (0.45 µm, Invitrogen, Carlsbad, CA, USA) with Trans‐Blot Turbo System (Bio‐Rad Laboratories) in accordance with the manufacturer's instructions. Immunoblot analyses were performed using human anti‐SUMO‐2/3 monoclonal antibody (1:2 000, MBL International) and chemiluminescence detection reagents (Pierce^TM^ ECL Western Blotting Substrate, Thermo Fisher Scientific). Quantitative analyses of chemiluminescence were performed using Image Lab software for a ChemiDoc instrument (Bio‐Rad Laboratories), with default settings (‘auto‐exposure’). To ensure that detected chemiluminescence signal was within the linear dynamic range (and thus to prevent signal saturation during the imaging), Image Lab software was set up to highlight saturated pixels (‘highlight saturated pixels’ function). None of our gels showed signs of oversaturation. Finally, SUMOylation status of each follicular fluid ‐treated sample of all the males was compared (standardized) to the control (i.e. non‐treated) samples of each individual males. In other words, SUMOylation status of the control samples was given a value 1 and reported SUMOylation values of the follicular fluid ‐treated sperm samples are presented in relation to this value. Accordingly, SUMOylation value of 0.5 indicates that such sample contains 50% less SUMOylated proteins than the control sample and similarly value 2 indicates two times higher SUMOylation signal in relation to control. This standardization allowed us to compare the relative influence of follicular fluid on sperm SUMOylation status across different gels and thus rule out the influence of gel‐specific exposure time differences on our results. Determined protein SUMOylation status was repeatable across replicate gels for both sample types (intra‐class correlation coefficient, sperm: 0.62, *p* = 0.001, *n* = 50; follicular fluid: 0.92, *p* = 0.017, *n* = 5).

### 2.5 Statistical analyses

The effects of male, female, male–female interaction and sperm SUMOylation status on sperm swimming velocity (VCL), hyperactivation (percentage of hyperactivated sperm cells) and sperm viability were tested in linear mixed‐effects models (LMM). The full model for sperm motility parameters included male, female and male–female interaction as random effects and sub‐sample as a fixed effect. Furthermore, since sperm motility traits were measured in four different time points, the full model also included time point as an additional (continuous) fixed effect. Due to model convergence problems, the slope of time point on above‐mentioned random effects (i.e. time point‐random effects interactions) could not be included in the full model. Thus, the effect of male, female, male–female interaction and sperm SUMOylation status on measured sperm traits was studied separately at each time point.

Similar to the full models, time point‐specific models for VCL, and hyperactivation, as well as sperm viability model also included male, female and male–female interaction as random effects and sub‐sample as a fixed effect. Furthermore, these models also included sperm SUMOylation status as an additional fixed effect (covariate). To test whether the slope of sperm SUMOylation was similar across different males, we also modelled the interaction between sperm SUMOylation and male (SUMOylation|male) as an additional random factor. Finally, we tested the effect of male, female and male–female interaction on sperm SUMOylation status (response variable). Initial model for sperm SUMOylation included replicate gel and follicular fluid SUMOylation status as fixed effects and following random effects: male, female and male–female interaction, as well as the interactions between replicate and male (1|replicate:male) and replicate and female (1|replicate:female). Based on AIC (−2 × (log‐likelihood—number of model parameters)), replicate–female interaction was removed from the final model (Table S1). Similarly, to test the effect of sperm incubation volume (110 µl–460 µl) on sperm SUMOylation status, VCL, hyperactivation and viability, we also included sperm incubation volume (sperm SUMOylation model) and the interaction between sperm incubation volume and SUMOylation status (models for VCL, hyperactivation and viability) as additional fixed factors in our models. Then, we simplified the models based on AIC. In all the models, these additional fixed factors did not improve model fit and were thus removed from the final models (Table S1). Model assumptions were graphically verified using Q–Q plots and residual plots. All *p*‐values presented are from two‐tailed tests, with *α* = 0.05. All the statistical analyses were conducted using the package lmerTest (Kuznetsova et al., 2017) in R (version 4.0: R Core Team, 2021).

## 3 RESULTS

### 3.1 Sperm motility and viability

Sperm swimming velocity (VCL) and the proportion of hyperactivated sperm cells decreased with time (time point: *p* < 0.001, in both cases, Table S2). Time point‐specific analyses revealed that both VCL (Table 1) and hyperactivation (Table 2) were affected by male in all four time points, whereas female effects were statistically significant in 30 min, 90 min and 300 min. Male–female interaction was statistically significant in 180 min and 300 min (and for VCL also in 30 min). Sperm viability was affected by all three random effects (male: *χ^2^* = 67.89, *p* < 0.001; female: *χ^2^* = 6.78, *p* = 0.009; male ×female: *χ^2^* = 13.78, *p* < 0.001).

**TABLE 1:** Linear mixed model statistics for the effect of male, female, male–female interaction (M × F) and sperm protein SUMOylation (SUMO) on sperm swimming velocity (VCL) in four different time points (30–300 min after the initiation of follicular fluid treatment)

Effects	30 min			90 min			180 min			300 min		
Random	*χ^2^*	df	*p*‐value	*χ^2^*	df	*p*‐value	*χ^2^*	df	*p*‐value	*χ^2^*	df	*p*‐value
Male	126.8	1	**<0.001**	121.8	1	**<0.001**	40.6	1	**<0.001**	35.3	1	**<0.001**
Female	6.7	1	**0.010**	13.5	1	**<0.001**	2.3	1	0.13	19.2	1	**<0.001**
M × F	5.1	1	**0.023**	0.1	1	0.76	28.9	1	**<0.001**	64.5	1	**<0.001**
SUMO|Male	–	–	–	–	–	–	13.06	2	**0.001**	–	–	–
**Fixed**	***t***	**df**	***p*‐value**	***t***	**df**	***p*‐value**	***t***	**df**	***p*‐value**	***t***	**df**	***p*‐value**
Intercept	15.8	10.5	**<0.001**	19.0	11.4	**<0.001**	23.1	19.0	**<0.001**	15.6	15.3	**<0.001**
Sub‐sample	2.6	155.9	**0.010**	4.5	156.8	**<0.001**	1.3	153.7	0.21	−0.5	151.8	0.65
SUMO	−0.3	177.3	0.76	−3.1	147.2	**0.002**	−3.3	194.1	**0.001**	−5.6	181.6	**<0.001**

We have bolded all the p‐values that are smaller than 0.05.

**TABLE 2:** Linear mixed model statistics for the effect of male, female, male–female interaction (M × F) and sperm protein SUMOylation (SUMO) on sperm hyperactivation in four different time points (30–300 min after the initiation of follicular fluid treatment)

Effects	30 min			90 min			180 min			300 min		
Random	*χ^2^*	df	*p*‐value	*χ^2^*	df	*p*‐value	*χ^2^*	df	*p*‐value	*χ^2^*	df	*p*‐value
Male	114.3	1	**<0.001**	109.7	1	**<0.001**	51.1	1	**<0.001**	43.1	1	**<0.001**
Female	9.2	1	**0.002**	10.5	1	**0.001**	3.4	1	0.064	15.0	1	**<0.001**
M × F	1.2	1	0.26	0.0	1	1.0	5.9	1	**0.016**	51.7	1	**<0.001**
SUMO|Male	–	–	–	–	–	–	16.54	2	**<0.001**	–	–	–
**Fixed**	***t***	**df**	***p*‐value**	***t***	**df**	***p*‐value**	***t***	**df**	***p*‐value**	***t***	**df**	***p*‐value**
Intercept	6.2	11.5	**<0.001**	7.6	12.3	**<0.001**	8.5	20.6	**<0.001**	5.8	17.1	**<0.001**
Sub‐sample	2.3	156.7	**0.021**	3.4	184.5	**<0.001**	0.7	156.1	0.51	−0.4	152.4	0.66
SUMO	−1.0	162.6	0.31	−2.4	190.2	**0.019**	−2.6	186.3	**0.010**	−5.0	184.7	**<0.001**

We have bolded all the p‐values that are smaller than 0.05.

### 3.2 Sperm SUMOylation status and associations between sperm traits

**FIGURE 1:**
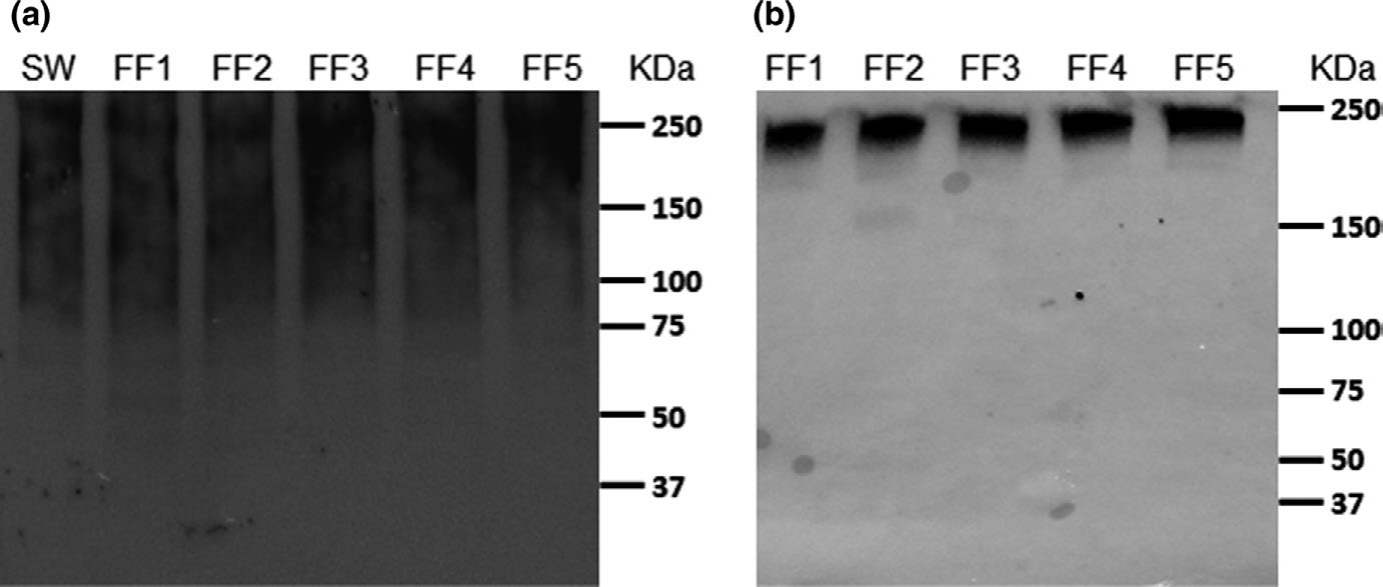
Western blot images for global sperm (a) and follicular fluid (b) protein 2/3 SUMOylation. Figure a shows SUMOylation patterns in one of the male subjects after five hours treatment with the follicular fluid of five females (FF1–FF5) and in a control sample (SW: Sperm Wash solution). Figure b shows SUMOylation patterns in the follicular fluids of the five female subjects. The positions of molecular weight standards (KDa) are indicated on the right side of the figures

**FIGURE 2:**
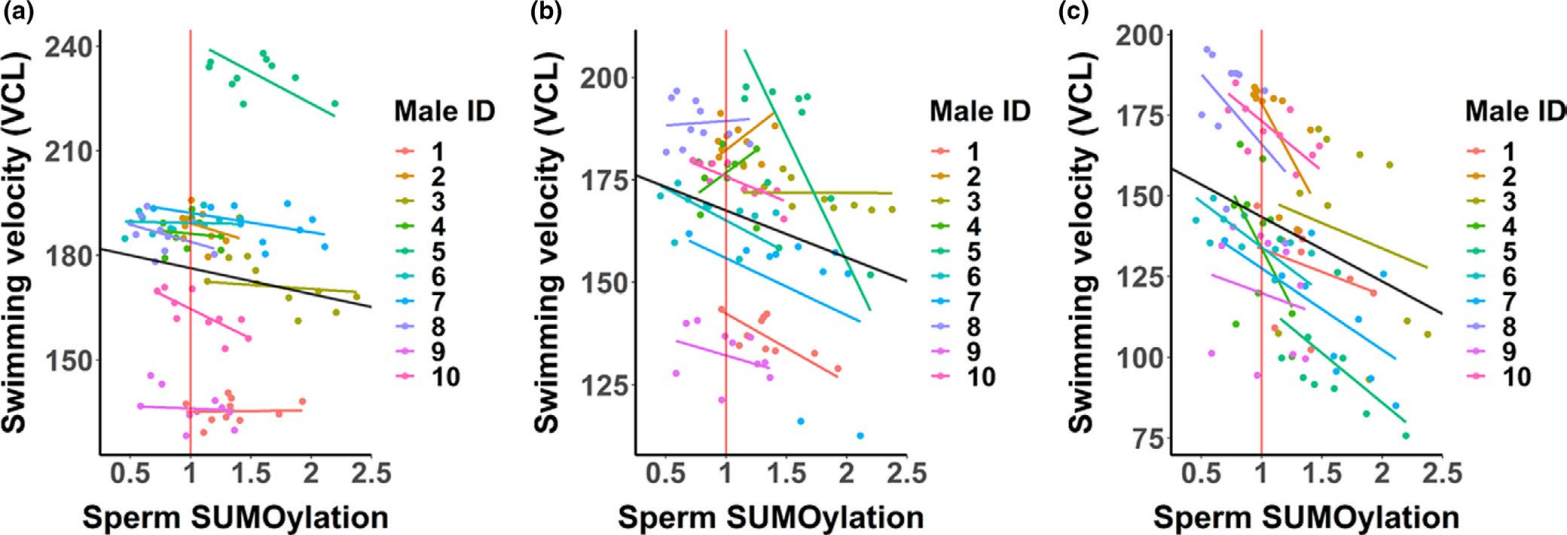
Association between sperm SUMOylation status and swimming velocity (VCL) 90 min (a), 180 min (b) and 300 min (c) after the initiation of sperm‐follicular fluid treatments. Datapoints represent fitted values obtained from the LMM. Red vertical lines indicate sperm SUMOylation status in the control samples of each of the 10 males (i.e. SUMOylation values are presented in relation to the control samples of each male). Male‐specific associations are identified by different colours and the black line represents the average slope across all males. The slope of the association differed between males in 180 min (*p* < 0.001), but not in 90 min (*p* = 0.10) and 300 min (*p* = 0.49)

**FIGURE 3:**
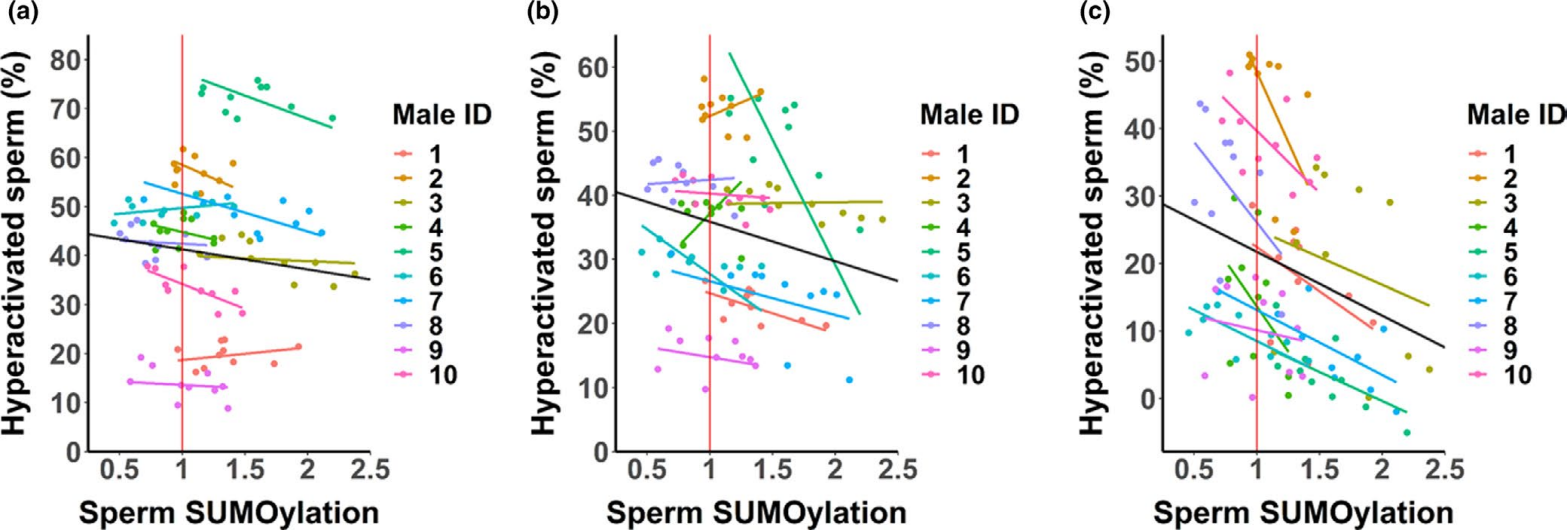
Association between sperm SUMOylation status and proportion of hyperactivated sperm 90 min (a), 180 min (b) and 300 min (c) after the initiation of sperm‐follicular fluid treatments. Datapoints represent fitted values obtained from the LMM. Red vertical lines indicate sperm SUMOylation status in the control samples of each of the 10 males (i.e. SUMOylation values are presented in relation to the control samples of each male). Male‐specific associations are identified by different colours, and the black line represents the average slope across all males. The slope of the association differed between males in 180 min (*p* = 0.001), but not in 90 min (*p* = 0.12) and 300 min (*p* = 0.25)

In sperm, protein SUMOylation was detected mainly in 75–250 kDa proteins, whereas in follicular fluid, all the SUMOylated proteins were relatively large (~250 kDa) (Figure 1). Sperm SUMOylation status was affected by male–female interaction, but the effect of male and female was not statistically significant (Table 3). Furthermore, follicular fluid protein SUMOylation status was not associated with sperm SUMOylation status. The effect of follicular fluid on sperm SUMOylation status was not consistent across different male–female combinations: In relation to control samples, follicular fluid not only increased sperm SUMOylation in most of the male–female combinations (Figures 2 and 3: Sperm SUMOylation > 1), but also frequently decreased it in the other combinations (Sperm SUMOylation < 1). Overall, SUMOylation of sperm was negatively associated with sperm swimming velocity and hyperactivation in last three time points (90, 180 and 300 min, Tables 1 and 2, Figures 2 and 3). The interaction between sperm SUMOylation and male was statistically significant for both VCL and hyperactivation in 180 min (VCL: *χ^2^* = 13.06, *p* = 0.001; hyperactivation: *χ^2^* = 16.54, *p* < 0.001), but not in 90 min (VCL: *χ^2^* = 4.52, *p* = 0.10; hyperactivation: *χ^2^* = 4.29, *p* = 0.12) or 300 min (VCL: *χ^2^* = 1.43, *p* = 0.49; hyperactivation: *χ^2^* = 2.79, *p* = 0.25). In other words, the effect (slope) of SUMOylation on sperm motility was similar across 10 males in 90 and 300 min but showed between‐male variation in 180 min (Tables 1 and 2). Sperm SUMOylation status was not associated with sperm viability (*t* = −0.02, *p* = 0.99).

**TABLE 3:** Final linear mixed model statistics for the effect of male, female, male–female interaction, replicate–male interaction and follicular fluid protein SUMOylation (FF SUMO) on sperm protein SUMOylation status

Effects				
Random	*χ^2^*	df	*p*‐value	% of total variance
Male	1.76	1	0.18	32.55
Female	0.08	1	**0.78**	0.47
Male × Female	8.14	1	**0.004**	11.10
Replicate × Male	39.37	1	**<0.001**	40.39
Residual				14.26
**Fixed**	***t***	**df**	***p*‐value**	**% of total variance (both fixed effects)**
Intercept	7.45	17.65	**<0.001**	
Replicate	0.66	9.57	0.53	
FF SUMO	0.37	4.72	0.73	
Total variance				1.23

We have bolded all the p‐values that are smaller than 0.05.

## 4 DISCUSSION

Present results show that follicular fluid modifies motility, viability and global protein SUMOylation status of the sperm and that all these effects are dependent on male–female combination. We also observed that sperm SUMOylation status is negatively associated with sperm motility, indicating that excessive SUMOylation may be detrimental for sperm fertilization capability. The effect of follicular fluid treatment on sperm SUMOylation status was found to be selective: Follicular fluid increased sperm SUMOylation in some male–female combinations but decreased it in the others. Together, these findings indicate that follicular fluid may selectively modify sperm protein SUMOylation and this way possibly mediate gamete‐mediated mate choice towards compatible or otherwise preferred males (see Fitzpatrick et al., 2020; Jokiniemi, Kuusipalo, et al., 2020; see also Jokiniemi, Magris, et al., 2020). We also found that the magnitude of male–female interaction effect and the strength of the association between sperm SUMOylation status and motility increased with time, indicating that selective recruitment of sperm for fertilization may require at least 3–5 h. Accordingly, our findings may offer novel insights into the molecular mechanisms of non‐random fertilization in humans (reviewed by Kekäläinen & Evans, 2018). However, due to the relative low amount female subjects and the fact that two females had been diagnosed with female‐factor infertility, some caution should be applied when interpreting female effects of our models and to generalize our findings to human population at large.

Fertilization is a complex molecular‐level signalling process and involves numerous direct and indirect interactions between male and female reproductive proteins (Carlisle & Swanson, 2020; Claw et al., 2014; Levitan & Ferrell, 2006). It has also been suggested that these proteins could determine the reproductive compatibility of the mating partners during fertilization (Hart et al., 2018; Rohlfs et al., 2010; Stapper et al., 2015; Vicens & Roldan, 2014). Furthermore, recent studies have demonstrated that females are able to favour the sperm of compatible males prior to physical contact of the gametes via egg and/or female reproductive tract‐derived chemical factors (Aguirre et al., 2016; Fitzpatrick et al., 2020; Gasparini & Pilastro, 2011; Geßner et al., 2017; Jokiniemi, Kuusipalo, et al., 2020; Jokiniemi, Magris, et al., 2020; Oliver & Evans, 2014; Yeates et al., 2009). However, the exact molecular mechanisms of such remote forms of cryptic female choice have remained unclear. Johnson et al. (2020) recently demonstrated in Chinook salmon (*Oncorhynchus tshawytscha*) that ovarian fluid proteins may play important role in this process. This raises a possibility that female‐derived reproductive secretions could control pre‐fertilization interactions between gametes and selectively favour the sperm of genetically compatible males. However, to the best of our knowledge, none of the earlier studies have investigated the protein post‐translational modifications in this context.

At least two non‐mutually exclusive mechanisms can explain observed non‐random patterns of sperm protein SUMOylation. During the migration in the female reproductive tract, sperm are immersed with various female reproductive secretions, which contain a wide variety of chemical factors, including nutrients, hormones, growth factors and proteins (Luongo et al., 2020; Machtinger et al., 2016; Soleilhavoup et al., 2016). These factors can trigger pre‐fertilization functional changes in sperm and can be transferred into the spermatozoa. Accordingly, it has been demonstrated that female reproductive secretions can be delivered into the sperm, which in turn could potentially directly shape the post‐translational modification status of the sperm proteome (Bathala et al., 2018; Fereshteh et al., 2019; Franchi et al., 2020). However, our results demonstrated that sperm SUMOylation status was not associated with follicular fluid SUMOylation status, indicating that the observed changes in sperm SUMOylation patterns may not be affected by follicular fluid mediated transportation of SUMOylated proteins into sperm. Alternatively, female‐derived chemical factors may activate signal transduction pathways in the sperm cells leading to the enzymatic reactions responsible for protein SUMOylation. Supporting this view, it has been shown that SUMOylation (and possible also de‐sumoylation) pathways can be activated in ejaculated sperm in response to the external stimuli (Marchiani et al., 2014, see also Yi et al., 2012, for de novo protein ubiquitination and de‐ubiquitination in capacitating sperm). Based on the above‐mentioned findings, it is likely that follicular fluid may be capable of selectively regulating these pathways in sperm. Therefore, female‐induced structural and functional modifications of sperm proteome may provide novel insights into the deeper mechanistic understanding of gamete‐mediated mate choice. However, since our study is based on the follicular fluid samples of only five females, two of which had been diagnosed with infertility, further studies utilizing larger subset of fully fertile women are required to confirm our results.

The primary aim of the present study was to investigate the influence of follicular fluid identity on global protein SUMOylation status of the sperm. Thus, detailed protein‐specific targets of demonstrated female‐induced SUMOylation process need to be clarified in further studies. However, it has been demonstrated that in ejaculated human spermatozoa, SUMO 1 and SUMO 2/3 are enriched in proteins in the ‘neck’ area of sperm and were also found in flagella and some sperm head regions (Vigodner et al., 2013). It has also been shown that the amount of SUMOylated proteins is higher in poor quality spermatozoa (Baker, 2016). Accordingly, non‐motile and morphologically abnormal sperm were found to have higher levels of SUMOylation than normal sperm (Vigodner et al., 2013) and SUMOylation is positively associated with sperm DNA fragmentation (Marchiani et al., 2014). Supporting these earlier findings, we found a strong negative association between follicular fluid‐induced global SUMOylation and sperm motility. Consequently, observed female‐induced selective changes in sperm protein SUMOylation status may have important role in mediating the fertilization bias towards the sperm of specific (‘selected’) males.

In conclusion, our results indicate that follicular fluid of the females is capable of selectively regulating the SUMOylation status of the sperm proteome and this way facilitates mate choice at the level of the gametes. Therefore, female‐induced post‐translational modifications in the structure and function of sperm proteins may constitute a novel mechanism of gamete‐mediated mate choice in humans. Furthermore, present results may have implications for the deeper understanding of infertility (Kekäläinen, 2021). Accordingly, along with male and female pathological conditions, fertilization failure may also arise from a gamete‐level incompatibility of the partners (see also Jokiniemi, Kuusipalo, et al., 2020; Jokiniemi, Magris, et al., 2020). Therefore, more comprehensive understanding of the mechanistic basis of demonstrated non‐random post‐translational modifications of the sperm proteome may open novel possibilities for the development of more accurate infertility diagnostics (see Brohi & Huo, 2017).

## CONFLICT OF INTEREST

The authors have no conflict of interest to declare.

## AUTHOR CONTRIBUTIONS

JK and MM conceived the study. LK, MH and JL collected the samples. JH, AJ and MM performed the experiments. JK analysed the data and wrote the manuscript. All authors approved the final version.

### PEER REVIEW

The peer review history for this article is available at https://publons.com/publon/10.1111/jeb.13980.

## Supporting information

Table S1‐S2

## Data Availability

The data that support the findings of this study are openly available in figshare at https://doi.org/10.6084/m9.figshare.17171030.v1
