# Supplementary material for: Female‐induced selective modification of sperm protein SUMOylation—potential mechanistic insights into the non‐random fertilization in humans
Source: J Evol Biol. 2022 Jan 19;35(2):254–64. doi: 10.1111/jeb.13980 (PMC9305144; doi:10.1111/jeb.13980)
Supplement: Supplementary file 1 — Table S1‐S2 [file JEB-35-254-s001.docx]

**Supplementary Table 1.** Akaike's information criterion (AIC) values for sperm SUMOylation models (1) and for the models testing for the effect of sperm incubation volume on sperm VCL (2-5), hyperactivation (6-9) and viability (10).

| **Model** | **AIC: Full model** | **AIC: Final model** |
| --- | --- | --- |
| 1. Sperm SUMOylation | 74.8 | 62.9 |
| 2. VCL 30 min | 1488.0 | 1475.6 |
| 3. VCL 90 min | 1510.5 | 1497.6 |
| 4. VCL 180 min | 1638.6 | 1628.7 |
| 5. VCL 300 min | 1654.9 | 1643.8 |
| 6. Hyperactivation 30 min | 1385.1 | 1371.2 |
| 7. Hyperactivation 90 min | 1389.2 | 1376.3 |
| 8. Hyperactivation 180 min | 1487.6 | 1475.6 |
| 9. Hyperactivation 300 min | 1408.0 | 1393.3 |
| 10. Sperm viability | 1257.9 | 1248.2 |

**Supplementary Table 2.** Overall linear mixed model (LMM) statistics for sperm swimming velocity (VCL) and hyperactivation in follicular fluid.

| **Effects** | **VCL** | | |  | **Hyperactivation** | | |
| --- | --- | --- | --- | --- | --- | --- | --- |
| **Random** | *χ^2^* | d.f. | *P*-value |  | *χ^2^* | d.f. | *P*-value |
| Male | 87.40 | 1 | **< 0.001** |  | 96.00 | 1 | **< 0.001** |
| Female | 28.56 | 1 | **< 0.001** |  | 27.12 | 1 | **< 0.001** |
| Male × Female | 0.00 | 1 | 1.0 |  | 0.00 | 1 | 1.0 |
| **Fixed** | *t* | d.f. | *P*-value |  | *t* | d.f. | *P*-value |
| Intercept | 25.95 | 12.9 | **< 0.001** |  | 10.89 | 12.2 | **< 0.001** |
| Timepoint | -18.06 | 784 | **< 0.001** |  | -18.49 | 784 | **< 0.001** |
| Replicate | 0.68 | 784 | 0.50 |  | 0.74 | 784 | 0.46 |
